# Research integrity in forensic anthropology

**DOI:** 10.1080/20961790.2021.1963515

**Published:** 2021-08-31

**Authors:** Douglas H. Ubelaker

**Affiliations:** National Museum of Natural History,Smithsonian Institution, Washington, DC, USA

##  

Research represents the driving force for change and advancement in the forensic sciences [1]. Of course, forensic science features casework and the application of our scientific knowledge to medicolegal issues. However, that scientific knowledge is largely based upon research conducted over decades.

Much research is stimulated by casework and the associated problems and issues that must be addressed. This is particularly true in the field of forensic anthropology in which the casework landscape has evolved rapidly in recent years. Just a few decades ago, forensic anthropologists mostly examined relatively complete skeletons in criminal cases or isolated discoveries in need of identification. Today these contexts are augmented by the analysis of mass graves in global human rights investigations, decedents in mass disasters and attacks of terrorism, and living juveniles in immigration and other legal issues. While relatively complete skeletons continue to need analysis, these are now supplemented with fragmentary evidence, bodies in an advanced state of decomposition and radiographs of the living. Anthropologists assist with conventional autopsies and in recovery operations. Demand persists for the anthropological report, but with increasing frequency anthropologists participate in more comprehensive reporting and court testimony [2].

These evolving contexts present methodological challenges for the forensic anthropologist. Anthropologists frequently find that published, accepted methods are not available to address the types of problems that must be resolved in the modern laboratory. The cases themselves and their inherent problems, each of which requires resolution, represent powerful stimuli for new research. In recent years, new research has involved thoughtful experimentation and analysis of recently acquired new collections. At times, approaches to novel methods can be found in other scientific disciplines. Three examples from my research provide a case in point.

The first relates to my over 40-year forensic career, during which time I have reported on nearly 1 000 cases, mostly at the request of the Federal Bureau of Investigation (FBI) in Washington, DC. In the early years of this consultation, cases primarily consisted of individual skeletons or major parts thereof. Toward the end of this period, cases shifted to include more fragmentary evidence resulting from fire, blast trauma and/or complex criminal activity. When small fragments were recovered and analyzed, traditional techniques were not available to distinguish bone or tooth from the many other particles that resemble these skeletal fragments and are commonly found at recovery sites. Conversations with colleagues revealed that the FBI had already developed a method using scanning electron microscopy/energy dispersive spectroscopy (SEM/EDS) to recognize different recovered materials. Through a research project, examples of bone and tooth in a variety of taphonomic conditions were added to the FBI database. The resulting new method could distinguish very small particles of bone and tooth from others and became an invaluable new analysis tool in casework [3].

The method described above allowed for recognition of small fragments of bone and tooth but it could not distinguish human fragments from those of other animals. Such recognition is extremely important in casework to allow species determination prior to any further destructive techniques (such as molecular ana­lysis). Again in the second example, extending my knowledge through conversations with colleagues and search of published literature in other scientific disciplines revealed a likely solution. Zoologists had deve­loped a method to use radioimmunoassay analysis to examine taxonomic relationships of animals. The method was developed for small fragments and was also effective on fossilized material. New research demonstrated that the method could distinguish human fragments from those of other animals and even indicate the animal species represented, if not human [4]. Once fragments were identified as bone or tooth, this new method allowed species classification, even from human fragments of great antiquity and poor preservation.

The third example relates to the estimation of time since death. Casework and research have demonstrated that many variables impact the process of postmortem change in both soft and hard human tissues. Ground water, soil acidity, presence of scavengers, constitutional factors, type and presence of clothing and containers, temperature, vegetation, and other factors all influence the timing and nature of postmortem alteration. Since many of these complex factors are unknown in forensic cases involving skeletal remains, estimation of the postmortem interval is very difficult from morphological observation alone [5]. Early in my own career, I felt confident estimating in general terms the postmortem interval from observations of tissue preservation. As more data emerged on the variables involved, however, it became clear that even general estimates were problematic.

Like many forensic anthropologists of my generation, I conducted research not only on forensic issues but also in bioarcheology. Working with archaeological samples, radiocarbon dating has provided a solid chronological framework for decades. In the past, forensic anthropologists have considered radiocarbon dating to be irrelevant for modern cases since in the few recent decades of medicolegal interest there has not been enough radiocarbon decay to measure effectively. However, when the radiocarbon laboratories report their analyses, they refer to years before present, with “present” representing the year 1950. This year reflects not only laboratory convention but also the fact that after 1950 the normally stable levels of radiocarbon in the atmosphere were drastically increased by atmospheric thermonuclear bomb testing. These augmented levels peaked around 1963 and then gra­dually decreased following cessation of atmospheric testing. It occurred to me and others that these augmented levels of atmospheric carbon-14 presented an opportunity to clarify the postmortem interval in these problematic skeletal forensic cases. If radiocarbon ana­lysis revealed the higher modern levels, then the individual must have been alive after 1950. Furthermore, research revealed that due to variance in the formation times of bones and teeth and variable turnover rates, analysis of different tissues within a single skeleton could reveal the approximate birth and death dates. Proper selection of tissues for analysis is critical and age at death is also a factor [6]. Although radiocarbon analysis has been available for many decades, we only now realize its potential to determine the postmortem interval in skeletal cases.

These three examples represent a small fraction of the robust research conducted by forensic anthropo­logists in recent years. Increasingly this vital research includes experimentation with human cadavers and/or animal surrogates, formation and analysis of documented collections of human remains and use of clini­cal data, including images and photographs of living persons. Although this research is welcomed, indeed critical to improving the practice of forensic anthropology, integrity must be maintained, and potential ethical issues must be properly addressed. The remainder of this article explores integrity and ethical concerns in this context. While this discussion focuses specifically on forensic anthropology, most issues and recommendations relate to other areas of forensic science, as well as to the diverse disciplines with whom we collaborate and learn.

## Objectivity

Research, analysis, and case presentations in forensic science must remain objective. This responsibility is especially important for forensic anthropologists since their work is frequently imbedded within teams of other specialists and law enforcement personnel. The mission of forensic examination consists of objectively applying our scientific methods in the analysis and interpretation of evidence to seek the truth. This process must avoid improper influence of others who have particular theories or points of view on individual cases, whether such influence stems from political, economic or other pressure. Forensic anthropology enjoys a sound theoretical foundation [7], but safeguards must be in place to protect objectivity.

The employment situation of the anthropologist and organizational location of the laboratory can prove important in this regard. Most of my forensic research and casework has been conducted at the Smithsonian Institution in Washington, DC. Although the Smithsonian Institution operates within the federal government of the US, it offers its researchers and curators considerable freedom to conduct their work without undue influence from supervisors and administrators. This academic environment offers an ideal opportunity for independence and objectivity.

Such independence may be more problematic for anthropologists working within law enforcement, ­medicolegal teams, or other more mission-oriented organizations. In some situations, the anthropologist may need to intentionally install safeguards to preserve individual research integrity and protect against improper influence.

Even for those working in independent laboratories, attention must be directed toward cases submitted by law enforcement or other agencies to limit bias. Some anthropologists insist that no contextual information be provided about the case being analyzed to limit any possibility of bias. More commonly, anthropologists will be provided only the information they need to know to properly analyze the forensic aspects of the case. The analyst must discourage any investigator who tries to convey theories or desired research results.

Most of my casework experience involved remains submitted by the FBI laboratory. From the beginning of my work with the agency we had an understanding that I would be provided only the basic information that I needed to know with each case. I have submitted numerous independent reports and cannot recall any instance of attempts to improperly influence my ana­lysis. When I have worked with other individuals and organizations on forensic issues, I have always made it a point to indicate at the outset my requirements to preserve objectivity.

I recall one case in particular involving human remains the FBI submitted to me for analysis. Although I found evidence of damage to the remains, it was not clear whether the alterations represented taphonomic factors sustained after death or if they could be linked to the death event itself. Years after submitting my report, I learned that local prosecutors were bringing charges against an individual, arguing that the altera­tions I had observed were produced by sharp force trauma leading to the death of the victim. Since my report did not conclude that sharp force trauma was involved, I was summoned to the pre-trial hearing by the defense. This case reveals that although the expert analysis may be requested by a law enforcement agency, the results may ultimately benefit the defense effort.

When considering employment with a new organization, the forensic anthropologist should discuss these kinds of issues. Consider the goals of the organization, especially in the global arena of human rights investigations. Can the integrity and objectivity of the forensic anthropologist be protected within the structure and goals of the organization? In most cases, these issues can be addressed but only if they are recognized early and explicitly discussed. Failure to secure proper safeguards can later produce tension and even conflict. In some circumstances, it may be useful to produce a document clarifying concerns and the safeguards that need to be constructed. Payment for forensic consultation is proper but should never be arranged on a contingency basis.

While most factors of potential bias can be recognized and limited, others may be more subtle. To limit cognitive bias, the anthropologist must safeguard against easily made assumptions that may be incorrect. These can relate to status as a criminal case, time since death, association with artifacts, elements of the biological profile and most other areas of anthropological analysis. At trial, the expert must not assume guilt or innocence of the defendant. While questions posed at trial may reveal the perspective of the lawyer, the expert’s answers must be anchored in objectivity. Even if the lawyer at trial is challenging the witness’ credi­bility and may try to lead the expert to give a particular response, it is important to try to stay objective and neutral. Some technology can reduce the chances of bias but does not completely prevent it. Sources of bias in addition to those discussed above include training and personal factors relating to the analyst and basic human nature [8].

Objectivity must also be protected in the research process. Research involves formation of a hypothesis and testing. The research design should ensure objective data collection and analysis that fairly tests the hypothesis. Analysis needs to include proper statistical treatment and reporting of results with appropriate indications of the probabilities and errors involved. In both research and case reports, the conclusions must be consistent with the data and/or bench notes.

Efforts should be made to limit typographical mistakes and other errors in case reports and research manuscripts. Wording should be carefully considered to accurately reflect content and intent. Years ago, I developed a practice of repeatedly proof-reading docu­ments with a co-author or colleague until no errors could be detected and no further word adjustments were required. Usually, this requires multiple proof-reading sessions. References should be drawn from original sources and arranged in the correct format of the intended publication or other targeted outlet.

## Collections

Forensic anthropology research is largely collection based. To develop and test methods and hypotheses, forensic anthropologists frequently rely on documented collections of human remains and related materials. Such documentation includes individual information relating to age, sex, living stature, ancestry, disease history, and other such variables. Collections usually consist of skeletonized human remains but can include images and similar clinical information. Research use of such collections involves ethical considerations and judgment on a variety of issues [9].

The acquisition of new collections and perhaps even the use of collections raises questions that require examination of legal and ethical issues. Were the remains legally acquired? What is the documentation based upon and what is the probability of accuracy? Was there proper informed consent in the acquisition? Are the remains subject to repatriation laws and/or institutional policies? What permissions are required to access remains and conduct research? Once research is conducted, what is the proper manner to report/disseminate results?

Research integrity related to collections use involves proper safety measures, minimizing damage to the collections and proper collection selection. Adequate protection must be provided in the field, laboratory and storage units against fire, flood, high wind, theft, and other similar threats. Safety is usually a minimal concern when working with skeletonized museum collections. Proper masking for dust inhalation protection and wearing protective gloves are common protective measures. More advanced protection may be required when working with fleshed remains or those in an advanced state of decomposition [10].

Even routine handling of collections can be destructive when bones and teeth are in contact with each other and with hard surfaces. Efforts should be made to minimize direct contact and, when possible, guard against contact with hard surfaces, using soft surface coverings, cork rings for placement of crania, etc. Care must also be taken in placing remains in storage containers, ensuring that remains are not damaged when applying lids or placing units into storage structures. The storage environment should be regulated for proper temperature and humidity control. In some cultural contexts, community consultation as far as treatment of remains may be appropriate.

Although obvious to most researchers, mention is due to proper selection of collections. The research process calls for hypothesis testing and choosing collections that are appropriate for the research design [11]. For example, testing a method of estimating age at death calls for use of a documented collection of individuals of known age, not a collection recovered from an archaeological context with only estimated ages. Proper collection selection can involve regional considerations, sample size, preservation issues and availability.

Considerable recent research [12] involves destructive analysis. Histological research, the developments of isoscapes, DNA analysis and many other new approaches all call for various forms of destructive sampling. For such sampling, proper permissions must be obtained, and destruction should be kept as minimal as possible. Institutional guidelines must be followed regarding documentation prior to sampling and disposition of residual material. If multiple samples are involved, care must be taken to limit inter-sample contamination. Safeguards against environmental contami­nation are especially important in molecular research.

Research and casework in forensic anthropology call for using the best and most appropriate methods avai­lable. Such use includes appropriate statistical analysis, proper incorporation of technology, and thoughtful research design. Conclusions must be consistent with results and accurately reflect the limitations of the methods used. Conclusions and reports must present correct levels of probability and error assessment to ensure trust in the results.

Some consideration must also be given to the involvement of families and communities in research endeavours. In recent years, the global anthropological community has moved toward greater inclusion of such perspectives in research, casework and issues relating to collections. Such perspectives can be essential in the acquisition, curation, research use and repatriation of collections. Forensic applications related to human rights and humanitarian action routinely involve the affected community and family-based organizations [13]. Such involvement reflects shared interests in the research process and can provide valuable insights. Of course, forensic science must also guard against improper influence and bias and seek to preserve objectivity.

## Training

Training in forensic anthropology can be challenging. Forensic anthropologists address a broad range of legal issues involving a multitude of methods and problems to solve. The field of forensic anthropology includes participation in search and recovery, excavation, docu­mentation, species determination, assessment of sex, age at death, time since death, living stature and ancestry, evaluation of taphonomic alterations, assessment of trauma and evidence of foul play and identification. The forensic anthropologist must be familiar with legal issues and courtroom procedures [14] and applications in related areas of forensic science. Analysis includes not only the traditional calipers, but also advanced statistics and use of computerized databases. Specialized techniques may be called for, such as histology, isotope analysis, radiocarbon analysis, facial approximation and craniofacial photographic superimposition. Those working internationally need to understand law and policy relating to humanitarian action and the investigation of human rights abuse [15]. Each of these areas of forensic anthropology practice presents a substantial field of published literature, history of application and complex methodology.

The well-trained forensic anthropologist needs to be competent in all the basic areas of application and at least have a working knowledge of the value of the specialized techniques [16]. University graduate programsme face challenges in providing adequate training in such diverse academic areas. Students entering these programmes must decide on a programme of course work and research that will provide the necessary background. Many of the training programs are anchored within university anthropology departments that also require coursework in social anthropology, linguistics, and archaeology. In addition, the forensic anthropology graduate student may desire coursework in other departments focusing on anatomy, statistics, and other areas of forensic science. Advanced training in writing, public speaking, grant preparation, finance management and report structuring can also bolster confidence and enhance career development. This vast array of options forces hard decisions on the academic path to follow. Integrity involves choosing the appropriate path given the opportunities available and career goals and then honestly projecting that training in forensic applications. For example, the forensic anthropologist with no training in excavation should reveal that lack of experience when presented with a recovery operation requiring such expertise. Mentors can also play a criti­cal role in guiding students as they navigate career decisions and understand the value of proceeding with integrity regardless of their chosen path.

In casework, or in any domain, the forensic anthropologist needs to recognize his/her academic limitations and disclose them, guarding against offering opinions beyond their expertise or in other areas of forensic science. A forensic anthropologist may be well qualified to render opinions on dental anatomy and dental age changes but should leave analysis of dental restorations to the forensic odontologist. Forensic anthropologists may find the evidence of trauma, but in most jurisdictions, forensic pathologists must determine cause and manner of death. This need for humi­lity and transparency also applies to clothing analysis, ballistics, soft tissue interpretation, legal issues and other areas of forensic science, or any field for that matter, in which other specialists are better positioned academically to render opinions.

Accurate recognition of training and expertise provides the foundation for organizational ethics requirements. For example, the code of ethics and conduct of the American Academy of Forensic Sciences (AAFS) states that an AAFS member or affiliate must not “materially misrepresent his or her education, training, experience, area of expertise, or membership status within the Academy”, as well as “data or scientific principles upon which his or her conclusion or professional opinion is based” [17]. While it may be easy to embrace such laudable declarations, some subtle nuances can be challenging, especially when under the duress of court testimony. Personal integrity can dictate the path forward in these situations.

## Authorship, plagiarism and peer review

Publications and final case reports represent central goals in forensic anthropology practice. Given their importance in the research process, they deserve special attention in efforts to preserve integrity. Authorship issues top the list of areas of concern. The author list should include all individuals who made intellectual contributions and exclude those who did not. The journal *Forensic Sciences Research* facilitates this decision-making process by requesting that upon manuscript submission, the contributions of each author be defined (available from: https://www.tandfonline.com/action/authorSubmission?show=instructions&journal-Code=tfsr20, accessed 25 July 2021). The *Journal of Forensic Sciences* also provides guidance on authorship, noting that data collection and general supervision of the research group do not qualify (available from: https://onlinelibrary.wiley.com/page/journal/15564029/homepage/forauthors, accessed 25 July 2021). The first author should be the person with primary responsibility for the manuscript content. Most journals agree that the order of authors listed should be decided and agreed upon by the authors themselves. The International Committee of Medical Journal Editors (ICMJE) offers useful guidelines on authorship and related scholarly publishing issues (available from: http://www.icmje.org/recommendations, accessed 25 July 2021).

Plagiarism represents a major integrity concern for authors and journals alike. As defined in the US regu­latory context, plagiarism consists of the “appropriation of another person’s ideas, processes, results, or words without giving appropriate credit” (available from: https://ori.hhs.gov/definition-research-misconduct, accessed 25 July 2021). This definition has been incorporated or adapted in codes and guidelines around the world. In essence, scholarly work must be original and not published elsewhere without disclosure and permission. If detected, plagiarism can land a near fatal blow to an individual’s integrity. Many journals use plagiarism detection software, and some declare the right to bar an author from future publication if plagiarism is detected. Authors must take care to cite the work of others and seek permissions to publish direct quotes of major length, as well as illustrations, whether published or presented. Scientific writing must exemplify respect and acknowledgment, giving proper credit to others whenever appropriate to do so. For authors who publish extensively on similar topics, self-plagiarism can emerge as an issue. Subtle self-plagiarism can usually be avoided with original text, illustrations, and tables, but concerned scholars can consult detection systems such as www.ithenticate.com.

Most journals also require disclosure of funding sources and ethics compliance in the use of human or animal subjects. Citing funding sources (and other interests that could give rise to a real or perceived bias) provides credit to those supporting projects but also reveals any potential influence of the source on the project goals and results. The ethics statements serve as useful reminders that rules may be in place for some projects and likely vary with different institutions.

Publishing trustworthy results in reputable journals enhances research integrity. In recent years, peer review has increased as an issue for scholars’ career advancement in forensic science. In most cases, the scholar’s home institution considers the extent of peer review in evaluating the scholar’s publication record. The impact factor of the journal has also grown in importance in this regard. Many journals in forensic science have formed recently, presenting a variety of publishing options but also highly variable systems of peer review and business approach. Selecting the most appropriate journal for publishing research results can be guided by an examination of each journal’s editorial board in addition to the journal’s impact factor. Advice from respected senior colleagues can also prove especially useful.

Some colleagues cringe at the idea of peer review, largely due to the often extensive amount of time involved and the fear of unwarranted critical review. Although at times I have shared the concern about the length of time required for review, I generally welcome peer review. Reputable journals send review articles to esteemed colleagues who usually offer criti­cal but positive advice on how to improve a manuscript. My own work has almost always been improved through the thoughtful suggestions of colleagues in peer review. Outside perspectives from a knowledgeable colleague should be welcomed and appreciated.

Integrity issues abound in relation to scientific publishing. Once receiving critical review, authors usually either respond positively with editing or defend their point of view. In cases of manuscript rejection or severe criticism, some authors may seek publication in another venue. In other scenarios, an author may have participated voluntarily in an edited volume or meeting symposium and then, following criticism, chooses to publish the contribution singly elsewhere. In such cases, is the author obligated to acknowledge the previous criticism? Stewart [18] raised this issue as early as 1946, revealing that few guidelines were available at that time. Today, many journals request permission of reviewers to transmit their comments to another journal if the manuscript is submitted elsewhere following review and rejection.

In sum, issues inherent to responsible authorship and publication, avoiding plagiarism, and engaging in peer review present vibrant opportunities for demonstrating one’s integrity in the context of forensic research.

## Mentoring

A commitment to integrity in training also involves giving back. Ideally, experienced forensic anthropologists share their expertise with students and prospective forensic specialists through formal training programmes, lectures, workshops, and advice. Involvement of students in casework and research represents a prime teaching opportunity. Such activity can be symbiotically beneficial to both the instructor and the students, provided that the experience is appropriately structured and clear expectations and safeguards are in place. Care must be taken to ensure that students do not engage in forensic activities beyond their expertise or violate any policy relating to casework.

Sharing expertise, both formally and informally, is particularly important in global casework and research. A forensic specialist may be invited to analyze and report on a particular case in a country different from their own. That experience can be rewarding and may contribute to the resolution of the case. The impact of his or her involvement, however, can be increased if the casework is supplemented by local capacity building through workshops or direct training in case analysis. Most specialists involved in global capacity building report learning as much as they teach through such engagement. Impact is greatly enhanced if relationships are forged and the specialist’s visit leaves local colleagues trained to bring improved forensic practice to bear on future cases. Given the global interaction made possible by the modern internet, international sharing of expertise, knowledge, methods, and data have become not only relatively common but essential in some cases and nearly always rewarding.

## Summary and conclusions

While integrity in forensic research remains a universal goal, it is difficult to precisely define. The above discussion reveals key elements of research integrity and offers suggested guidelines. However, the individual forensic anthropologist must find their own way, given their training and work environment, as they navigate the ethical issues involved.

Modern forensic anthropologists need to preserve objectivity in their research and casework and take measures to limit bias. In report writing and court testimony, the forensic anthropologist must stay within their domain of expertise and avoid misrepresentation of their credentials and data. Effort should be made to limit errors in report writing and publications. Ethics statements of forensic organizations provide useful guidelines and should be shared and discussed with students.

Integrity in forensics research includes proper use of collections, especially regarding safety issues. Effort must be made to limit and carefully select any destructive analysis. Collection conservation procedures must be followed to limit damage to collections through use. Appropriate selection of collection samples for research must be made to match the research goals. Similarly, thoughtful selection of research methods contributes to research integrity.

Authorship deserves special attention, giving proper credit to those intellectually engaged in the project and writing. Plagiarism, including self-plagiarism, must be avoided through careful citation, and obtaining needed permissions. Forensic scholars must value peer review, giving proper responses if being reviewed and being fair if reviewing the work of others.

Community involvement and culturally sensitive communication represent important components of both research and casework, especially in global human rights work. Whenever possible, training and mentoring others and capacity building should be encouraged as important goals.

The forensic anthropologist conducting research and casework with integrity enjoys substantial professional rewards. Conducting a successful research process culminating in publication in a peer-reviewed journal or book represents a joyful professional experience. Such research establishes new methods, tests existing methods, and brings new technology to bear on looming forensic problems. Activity in forensic anthropology offers the reward of helping to resolve key issues of contemporary society.

Fortunately, many gifted and highly qualified students are attracted to the field of forensic anthropology. Today, these prospective forensic anthropologists find many educational opportunities and a growing, favourable employment environment. As they explore these opportunities and gain expertise, they must also focus on building credibility and maintaining integrity, keeping abreast of emerging technologies and ethical issues while remaining true to their personal and professional values.
